# A stepped-wedge implementation and evaluation of the healthy active peaceful playgrounds for youth (HAPPY) intervention

**DOI:** 10.1186/s12889-018-5397-6

**Published:** 2018-04-20

**Authors:** Dean Dudley, Wayne Cotton, Louisa Peralta, Matthew Winslade

**Affiliations:** 1 Department of Educational Studies, Macquarie University, North Ryde, NSW 2109 Australia; 2 Sydney School of Education and Social Work, The University of Sydney, Sydney, 2006 Australia; 3 School of Teacher Education, Charles Sturt University, Bathurst, 2795 Australia

**Keywords:** Physical activity, Social support, Student behaviour, SOPARC

## Abstract

**Background:**

Increasing physical activity in children is a health priority. The Healthy Active Peaceful Playgrounds for Youth (HAPPY) study aimed to examine a multi-component playground intervention designed to increase the proportion of physical activity during recess and lunch of primary school students.

**Methods:**

The 2016 Australian focused HAPPY Study was a 12-month, metropolitan primary school based intervention, which was evaluated using a stepped wedge design. The intervention combined teacher development, environmental modifications, and peer support, with the outcomes of increasing physical activity, and analysing students’ sources of social support to be physically active.

**Results:**

Between baseline and follow-up, the proportion of students involved in vigorous activities during recess and lunch times increased significantly from 15 to 25% (*p* < 0.001). No differences were recorded in sources of social support.

**Conclusions:**

The HAPPY project demonstrated an increase in the proportion of physical activity intensity during recess and lunch times in primary schools, although further investigation is required to determine what components of the study had the greatest effect.

**Trial registration:**

This study was retrospectively registered with the Australian and New Zealand Clinical Trials Registry ACTRN12616000575437. Date of registration: 4 May 2016.

## Background

There is strong evidence that physical activity (PA) is associated with a large number of physical health benefits [1, 2], as well as increased cognitive function [3], academic achievement [4, 5], and mental health [3, 6]. Unfortunately, international prevalence data suggest few children and adolescents are sufficiently active at the intensity and duration required to obtain these benefits [7]. This also includes Australian primary school-aged children, with current research suggesting that less than 1 in 4 (23%) are meeting the National Physical Activity Recommendations of 60 min a day of moderate to vigorous physical activity (MVPA) [8].

The school setting has the potential to provide children with opportunities to be physically active through a multi-sector, multi-system, multi-faceted approach [9, 10], as they have access to youth and often possess the facilities, equipment, and personnel required to deliver PE curricula, school sport and other non- and extra-curricular programs [10]. Despite showing promise, evidence suggests that schools have not been successful in promoting sufficient PA. For example, physical activity levels in these settings are typically much lower than recommended [11], and a considerable proportion of teachers, particularly primary school teachers, are not receiving the training needed to confidently deliver active, engaging and educative physical activity experiences [10, 12]. In addition, schools have been facing increasing demands for their student body to achieve academic goals, which may encourage schools to allocate less time for PE and school sport and redirect this to literacy and numeracy curriculum-based classroom activities [13]. Therefore, rather than loading teachers with increasingly more curricular responsibilities, providing them with knowledge and skills to promote MVPA during school break times and supervision is an important consideration [14].

Alongside PE, non-curricular periods, including recess and lunch breaks, are typically the domains with the highest proportion of time spent in MVPA during school hours [15, 16]. Hence, recent studies have sought to address the limited time for MVPA during the school day by focusing on enhancing MVPA among students during these break times [17–19]. The results of a recent systematic review suggests that a playground redesign, which utilizes multi-colour playground markings and physical structures including moveable, unfixed equipment, and identifying ways to promote encouragement for physical activity can have the potential to increase children’s break time PA [20]. However, the finding related to social support and encouragement for children’s break time PA was based on one study conducted in New Zealand with adolescents [21]. A subsequent study conducted in New Zealand which focused on primary schools, children’s break time and social connectedness found that increasing opportunities for risk and challenge (eg, rough-and-tumble play), reduced rules, and adding moveable equipment improved happiness at school and the amount of children playing with each other (as reported by the students themselves) [22]. As there is limited research that implements all of the systematic review’s suggestions in a primary school intervention focusing on increasing MVPA in school break times, this study focuses on combining teacher development, environmental modifications, and peer support, with the aim to increase physical activity, and analysing students’ sources of social support to be physically active.

## Methods

### Study design and participants

The Healthy Active Peaceful Playgrounds for Youth (HAPPY) Study was a 12 month, primary school-based intervention, which was evaluated using a cluster controlled trial with a stepped wedge design. Ethics approval was obtained from an Australian University Human Ethics Committee (HREA5201500676) and from the New South Wales (NSW) Department of Education and Communities (NSWDEC) (SERAP2015423). Further details about the study’s stepped wedge design, sample size, recruitment, participation, intervention design, statistical analysis (including power calculations) and main outcomes are readily available through an open access journal [23].

In brief, six NSWDEC schools in the Metropolitan Sydney Region were recruited to participate in the study. The study focused on evaluating the success of an intervention combining teacher development, environmental modifications, and peer support, with the outcomes of increasing physical activity, and analysing students’ sources of social support to be physically active. All six schools participated in baseline data collection during the school term 1, 2016 before being randomised into stepped intervention groups. Due to the nature of the intervention, school staff and students were not blinded to group allocation.

**Fig. 1:**
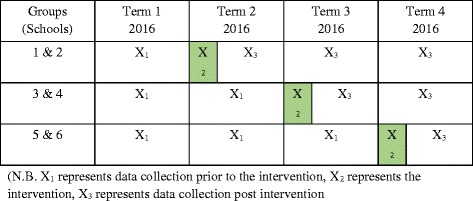
An overview of the study’s stepped wedge design

A power analysis was conducted in the protocol paper [23] and reported that the stepped wedge design used in this study would require 1661 students to be observed in order to provide adequate power to detect differences of *p* > 0.05 between baseline and follow-up. A total number of 20,468 students were observed across 713 iSOPARC scans at baseline (*n* = 115) and follow-up (*n* = 599). It is important to note that this does not represent individual students as the same students may be picked up in multiple scans, on multiple days (Fig. 1).

### Intervention

The HAPPY Study implemented the Peaceful Playgrounds Program [24] as the intervention. The Peaceful Playgrounds Program is a primary school-based package that uses a combination of teacher training, permanent playground markings and accompanying resources to promote physical activity in playgrounds, teach fundamental movement skills, and support social skill development in students.

The teacher training included strategies for initiating play during school breaks. These games and activities focused on the development of social and methods of supporting conflict resolution in the playground. The training also included access to an online network designed to aid teacher self-reflection and to provide opportunities for teachers to interact with other participating teachers and the research team.

The installation of multi-coloured permanent playground markings were used to act as visual prompts for students and teachers to be physically active. The markings used were specifically designed in conjunction with each participating school through a series of discussions with school executive and student groups. Teaching resources and moveable equipment (e.g., balls, skipping ropes), including activity suggestions and units of work related to the markings were also provided to the schools to support engagement with the playground markings.

Selected students from Stage 3 (Years 5 and 6) classes at each school also undertook a Student Leadership Program to develop knowledge and skills to become physical activity ambassadors during break times. These students were taught techniques from the Peaceful Playgrounds Program [24] to initiate games and encourage social inclusion with their peers.

### Data collection and measures

#### Physical activity during recess and lunch

The primary outcome variable for this study was the intensity of PA levels during recess and lunch breaks as a mean percentage of time available during those periods. The researchers were most interested in mean proportion of students engaging in vigorous physical activity (%VPA), Walking (%Walking), or sedentary activity (%sedentary activity). This was measured using the System for Observing Play and Recreation in Communities (iSOPARC) (CIAFEL, Portugal: https://ciafel.fade.up.pt/isoparc/) with simultaneous observational scans conducted by two research assistants trained to the gold standard from recorded and live observations.

A scan in iSOPARC involves panning from left to right, and coding each student in the area by sex (male or female), intensity of activity (Sedentary, Walking or Vigorous), and whether they are a Child, Teen, Adult or Senior. Additional data recorded for each scan included; 1. Time of day the observations were collected, and whether this was a lunch or recess period; 2. What type of activity the students are engaged in (i.e. handball, football, dancing, clapping games) and; 3. Teacher supervision and direction of physical activity. The SOPARC tool has been used by this research team to collect physical activity and sun-safe behaviours in primary schools in a previous study and was found to have a high degree of reliability with an Intraclass Correlation Coefficient (ICC) of .912 [25].

#### Sources of social support to be physically active

To measure the effects of the teacher training and student ambassador components of the intervention the Physical Activity Social Support Scale [26] was given to all students in Stage 3 (Years 5 and 6) classes at baseline and again at follow-up. The 15 min, nine question survey seeks information about how often students receive encouragement to be physically active and from whom. Test-retest reliability of the scale is strong (ICC = .88) and the Internal consistency of the items was evaluated at α = .77 [27].

#### Procedures

A University Human Research Ethics Committee approved the procedures for this study. Information sheets about the study were provided to all participants and their parents or legal guardian provided written informed consent. The My School website (https://www.myschool.edu.au/) provides publically available demographic data collected by the Federal government (e.g., the Index of Community Socio Educational Advantage—ICSEA, cultural background and language spoken at home).

Two trained research assistants administered and conducted the SOPARC observations, and informed classroom teachers administered the Physical Activity Social Support Scale.

#### Data analysis

Statistical analyses of the data collected was conducted using the Statistical Package for Social Sciences (SPSS, v21). The stepped wedge design of the study assumed that the delay in the intervention roll out to each school acts as the control period of the study. Therefore, means and standard deviations were calculated at baseline period prior to intervention and first follow-up point in the study for each of the respective groups. Each school received the intervention training over a two-week period that was then followed by eight weeks of follow-up observations. Test of normality were conducted using the Shapiro-Wilk test, and as the data was skewed, Mann-Whitney U tests were implemented to determine if there were any statistical difference between baseline and follow-up observations. Effect sizes were also calculated between the means at baseline and follow-up using Cohen’s d [28].

## Results

As planned, six New South Wales Department of Education and Communities (NSWDEC) schools from the Metropolitan Sydney region agreed to participate in the study. However, one of the six schools did not return adequate numbers of informed consent from participants and their guardians to allow their continuation in the study. This occurred because the liaison teacher retired from their position at short notice and did not make the forms that had been collected available to the researchers before his departure.

### Demographic data

An overview of the demographic characteristics of the five remaining schools in the study is reported in Table 1.

**Table 1:** Demographic characteristics of the students in the five schools involved in the study

	Baseline
Distribution of school students stratified by the ICSEA	
Bottom quarter	35.2%
Middle two quarters	52.8%
Top quarter	11.8%
Total student enrolment in schools	*n* = 2438
By sex	
Boys	*n* = 1296
Girls	*n* = 1142
Full-time equivalent teaching staff	156.9
Proportion of indigenous students	7.4%
Proportion of students from language backgrounds other than English	27%

Of the remaining five schools, when the students were stratified by the Index of Community Socio-Educational Advantage (ICSEA), a disproportional percentage of enrolled students (35.2%) were in the bottom quarter of the Australian population, with only 11.8% being in the top quarter. There were also a relatively high proportion of Indigenous students and students from language backgrounds other than English in the participating schools when compared with state averages.

### Physical activity during recess and lunch

Two trained research assistants collected data at both baseline and follow-up. Data was collected over 18 days during baseline and 79 days at follow-up. Interrater reliability was also tested at baseline, when both research assistants completed the same observations 36 times. From this data, a good ICC of .808 was calculated.

Table 2 reports the means of proportion of activity intensity (%) and standard deviations (SD) during recess and lunch breaks during recess and lunch breaks at baseline and follow-up. Only 115 observations were possible at baseline across the five schools due to the limited research assistants available and the complexity of the stepped wedge data collection design. The research team therefore prioritised data collection immediately after intervention delivery was made in most cases. Baseline data was collected from each of the five school nonetheless.

**Table 2:** Means of proportion of activity intensity (%), standard deviations (SD) and number of scans made (n) during recess and lunch breaks

Category	Recess & lunch behaviour (%)			Recess & lunch behaviour (%) Boys Obs.			Recess & lunch behaviour (%) Girls Obs.		
	Baseline M (SD) (*n* = 115)	Follow-up M (SD) *n* = 599)	Mean Diff. Cohen’s *d* (*p*-value)	Baseline M (SD) (*n* = 59)	Follow-up M (SD) (*n* = 299)	Mean Diff. Cohen’s *d* (*p*-value)	Baseline M (SD) (*n* = 56)	Follow-up M (SD) (*n* = 300)	Mean Diff. Cohen’s *d* (*p*-value)
Intensity of Physical Activity (via SOPARC)									
Sedentary activities	57.5 (27.6)	49.8 (30.5)	−7.7 0.26 (0.01)	52.5 (28.6)	42.9 (30.1)	−9.6 0.3 (0.01)	62.8 (25.8)	56.8 (29.2)	−6.0 0.2 (0.183)
Walking	26.6 (20.1)	25.1 (18.3)	1.5 0.1 (0.4)	26.8 (18.5)	23.2 (15.8)	−3.55 0.2 (0.1)	26.4 (21.8)	26.9 (20.3)	+ 0.5 0.02 (0.8)
Vigorous activities (VPA)	15.0 (21.8)	25.1 (26.6)	+ 10.1 0.4 (< 0.001)	19.0 (25.1)	33.9 (27.6)	+ 14.85 0.6 (< 0.001)	10.8 (16.9)	16.3 (22.4)	+ 5.5 30.3 (0.03)
	Recess & lunch behaviour (VPA%)								
Physical Activity observations (via SOPARC)	M (SD) Baseline	M (SD) Follow-up	Mean Diff. Cohen’s *d* (p-value)						
Hard Surface (*n* = 54 Baseline; *n* = 238 Follow-up) (i.e., Cement or Asphalt	8.7 (18.9)	13.7 (18.8)	+ 5.05 0.3 (0.004)						
Soft Surface (*n* = 60 Baseline; *n* = 361 Follow-up) (i.e., grass)	20.9 (22.8)	32.5 (28.4)	+ 11.6 0.4 (0.004)						

(N.B. As the data was non parametric, Mann-Whitney tests were applied)

Over the course of the intervention there was a significant decrease in sedentary activities (*p* < 0.05) and a significant increase in vigorous activities (*p* < 0.001). This increase was larger in boys. There was also a significant (*p* < 0.05) increase in the number of students being vigorously active on hard surfaces (i.e., cement or asphalt) and an even greater significant (*p* < 0.05) increase in students being vigorously active on soft surfaces (i.e., grass).

Table 3 reports the mean proportion of vigorous activity (VPA%) and standard deviations by sex and activity type. There was a significant increase in VPA% when students were engaged in a soccer context by both boys (*p* < 0.01) and girls (*p* < 0.05). There was also a significant (*p* < 0.01) increase in the number of boys participating in vigorous activity when engaged in a handball context.

**Table 3:** Mean of proportion of vigorous activity (VPA%) and standard deviations (SD) and number of scans made (n) by sex and activity type

Context of Physical Activity observations (via SOPARC)	Recess & lunch behaviour (VPA%)		Boys recess & lunch behaviour (VPA%)		Girls recess & lunch behaviour (VPA%)		Between group difference for Boys and Girls (95% CI)	
	Baseline	Follow-up	Baseline	Follow-up	Baseline	Follow-up	Baseline	Follow-up
	M (SD) ^n^ (*n* = 114)	M (SD) ^n^ (*n* = 599)	M (SD) ^n^ (*n* = 58)	M (SD) ^n^ (*n* = 299)	M (SD) ^n^ (*n* = 56)	M (SD) ^n^ (*n* = 300)	Mean Diff. (*p*-value)	Mean Diff (*p*-value)
Aerobics/Dance	50.0 (6.4) ^2^	41.0 (18.8) ^9^	N/A	N/A	50.0 (6.4) ^2^	41.0 (18.8) ^9^	N/A	N/A
Basketball	100 (N/A) ^1^	18.6** (20.6) ^28^	100 (0) ^1^	30.5** (19.4) ^16^	N/A	2.8 (7.0) ^12^	N/A	27.7 (< 0.01**)
Rugby/Touch Football	18.2 (N/A) ^1^	57.9 (23.6) ^34^	18.2 (N/A) ^1^	55.3 (22.0) ^31^	N/A	84.8 (26.2) ^3^	N/A	29.5 (< 0.18)
Handball	16.6 (15.7) ^14^	33.4** (22.2) ^56^	15.5 (16.2) ^10^	35.8** (22.0) ^50^	19.3 (16.6) ^4^	14.1 (12.8) ^6^	3.8 (< 0.71)	21.6 (< 0.01^*^)
Soccer	39.8 (21.2) ^22^	56.9** (19.1) ^125^	42.9 (21.3) ^17^	55.9* (18.0) ^93^	29.3 (19.0) ^5^	59.9* (22.1) ^32^	13.6 (< 0.21)	3.9 (< 0.36)

N.B. ^**^ Statistically significant at *p* < 0.01

^*^Statistically significant at *p* < 0.05

^n^Number of scans observing physical activity context

### Sources of social support to be physically active

Table 4 reports the results from the Physical Activity Support Scale. There were no statistically significant differences between groups for any of the subscales. Nor were there any effect sizes greater than *d* = 0.10. When analysed by sex, there were still no meaningful or statistical differences between the baseline and follow-up data on any of the subscales.

**Table 4:** Unadjusted means and standard deviations for the Physical Activity Support Scale at baseline and follow-up

Family and Peer Support Index	Baseline M (SD) (*n* = 401)	Follow-up M (SD) (*n* = 401)	Mean Diff.	*P*-value	Cohen’s *d*
Social Support (Adults)					
PA encouragement					
Male adult	2.1 (1.5)	2.3 (1.5)	0.2	0.211	0.09
Female adult	2.3 (1.4)	2.4 (1.5)	0.1	0.264	0.07
Other children	2.0 (1.5)	2.0 (1.5)	0.0	0.985	0.01
Participated in PA with participants					
Male adult	2.2 (1.4)	2.2 (1.4)	0.0	0.817	0.02
Female adult	2.1 (1.3)	2.1 (1.3)	0.0	0.785	0.02
Other children	1.7 (1.7)	1.7 (1.8)	0.0	0.980	0.01
Provided transportation for PA					
Male adult	2.2 (1.4)	2.3 (1.4)	0.1	0.445	0.04
Female adult	2.2 (1.5)	2.2 (1.5)	0.0	0.852	0.02
Other children	1.3 (0.9)	1.4 (1.0)	0.1	0.189	0.10
Watched participants participate in PA					
Male adult	2.3 (1.4)	2.2 (1.5)	−0.1	0.717	0.03
Female adult	2.3 (1.5)	2.2 (1.5)	−0.1	0.595	0.02
Other children	1.8 (1.4)	1.9 (1.5)	0.1	0.775	0.03
Told you that PA is good					
Male adult	1.9 (1.6)	1.9 (1.6)	0.0	0.703	0.04
Female adult	1.9 (1.6)	2.1 (1.6)	0.2	0.282	0.08
Other children	1.7 (1.3)	1.6 (1.3)	−0.1	0.569	0.03
Social Support (Peers)					
Encourage friends’ PA	2.3 (1.4)	2.2 (1.5)	−0.1	0.392	0.07
Friends encourage your PA	2.2 (1.4)	2.2 (1.4)	0.0	0.960	0.01
Friends do PA with you	1.7 (1.7)	1.7 (1.8)	0.0	0.731	0.02
Friends tease you about not being good at PA*	3.3 (1.1)	3.4 (1.1)	0.1	0.798	0.05

N.B. As the data was non-parametric, Mann-Whitney tests were implemented. Scale. 0 = none or don’t know, 1 = once, 2 = sometimes, 3 = almost daily, 4 = daily *Scale for this question reverse-coded (0 = none or don’t know, 4 = once, 3 = sometimes, 2 = almost daily, 1 = daily)

## Discussion

The Healthy Active Peaceful Playgrounds for Youth (HAPPY) study aimed to examine a multi-component playground intervention designed to increase the proportion of physical activity intensity among primary school students. Overall, the study significantly decreased the proportion of primary school students’ sedentary activity and significantly increased the proportion of student VPA significantly from 15 to 25%. In addition, there was a significant increase in the VPA% on both hard and soft surfaces, with the increase larger on soft surfaces. Notably, students who were vigorously active were engaged in the soccer or handball context.

These results are supported by other break time multi-component interventions, as published in systematic reviews [29, 30], which have found that the strategies that combined playground markings, playground coding or court rotation (to rotate playground use) and non-fixed equipment increased recess PA significantly, suggesting that these may be promising strategies that could benefit PA levels during school recess. However, a clear challenge of multi-component interventions is determining which of the strategies has the most impact on PA. Previous studies, including this study, have used a range of different strategies, making it difficult to conclude which approach is most effective. However, what this study does add is the locations and contexts where students were vigorously active.

By using an observational tool, the study found that %VPA for both girls and boys during recess and lunch break times significantly increased; however there was a larger increase in boys %VPA compared with girls. This positive finding has not always been represented in other break time studies. For example, previous research indicates that boys prefer to play games, including ball, fantasy and rough and tumble games [30–32] with boys tending to dominate playground space and play in larger groups [31, 32]. As a result, girls shy away from this domination and are often on the periphery interacting in smaller groups [33]. Also, another study found no differences in objectively measured physical activity levels of both boys and girls [19]. As such, it is surprising that this study’s results contradicts previous research with the intervention being able to significantly increase girls and boys %VPA. In addition, this study further explored the contexts in which students were being vigorously active and found that both hard and soft surfaces provided opportunities, with the largest increase experienced on soft surfaces. This suggests that physical activity promoting strategies should direct extra resources such as markings, teacher supervision and equipment to soft surfaces to ensure that both boys and girls experience as many opportunities as possible to be vigorously active.

Similar to the work of Pellegrini [34] and Powell et al. [35], students in this study enjoyed games that were collaborative, yet competitive, and governed by clear rules and boundaries that for the most part were known to all students. Research has shown that sports with set rules often limit play behaviours, mainly due to the power hierarchies that occur within the break time environment, which can be affected by school policies such as playground boundaries, teacher supervision and children’s social interactions [36]. Interestingly, the social support results of this study were not significant, which may support previous research that has shown that when students engage in break time sports, with set rules and boundaries, social hierarchies through physical prowess become prominent and general socialisation and support is limited [22, 37].

### Strengths and limitations

The use of direct observation allowed for the measurement of contextually rich data and is a method which is believed to exceed other PA measures [38]. One of its major strengths is the ability not only to measure PA levels but also the identification of the type of activity, when, where and with whom it occurs [38].

Another strength was the advantage afforded by use of a stepped wedge design that allowed the intervention to be rolled out to all participants and therefore motivating students and teachers to participate in this study. The limitation of the stepped wedge design however was the increased the complexity of the data analysis, and there were concerns regarding the informed consent procedure in one school that had to be removed from the study.

There are numerous limitations with this study. First, uneven sample sizes, the different numbers of repeated measures across stepped wedge, and the data collected through SOPARC (not individual student-based) all contribute to challenges with analysis and reporting of findings. Another limitation of the study would be the collection of data within one metropolitan area of New South Wales, Australia, which could affect the external validity of the study. Despite this, it should be acknowledged that the labour-intensive nature of direct observation limits the number of samples that can be taken [37]. Yet the consistency of the findings across the four schools suggests that the results could be shared with schools of similar demographics.

Another limitation presented in this study is that the iSOPARC tool may provide context for analysis not available in other objective measures (i.e. accelerometry) but is limited by its momentary analysis of activity intensity. This is because SOPARC requires the observer to conduct scans of the observation area and code the intensity type in quick succession. The dynamic nature of children in a school playground wearing similar uniforms means that the possibility of double coding an individual may occur. This limitation is exacerbated with larger observation areas and more students. However, the researchers felt these limitations were offset by the fact that SOPARC assisted researchers by providing a reliable, efficient and user-friendly means of data collection.

## Conclusion

The HAPPY study was successful in increasing the proportion of vigorous physical activity intensity of primary school-aged girls and boys during recess and lunch breaks. In particular, girls and boys were proportionally more active on soft surfaces and in soccer and handball contexts. Future research needs to investigate what components of multi-component interventions have the most impact on the proportion of physical activity intensity of girls and boys and to determine strategies that increases social support to be physically active during school break times.

## Abbreviations

ACTRN: Australian Clinical Trials Registry Number
CT: Controlled Trial
HAPPY: Healthy Active Peaceful Playgrounds for Youth
HREA: Human Research Ethics Application
ICC: Intra-cluster correlation
MVPA: Moderate – Vigorous Physical Activity
NSW: New South Wales
NSWDEC: New South Wales Department of Education and Communities
SERAP: State Education Research Applications Process
SOPARC: System for Observing Play and Recreation in Communities
SPSS: Statistical Package for the Social Sciences
